# Remote constraint induced therapy of the upper extremity (ReCITE): A feasibility study protocol

**DOI:** 10.3389/fneur.2022.1010449

**Published:** 2022-11-18

**Authors:** Lauren J. Christie, Nicola Fearn, Annie McCluskey, Natasha A. Lannin, Christine T. Shiner, Anna Kilkenny, Jessamy Boydell, Annie Meharg, Ella Howes, Leonid Churilov, Steven Faux, Arlette Doussoulin, Sandy Middleton

**Affiliations:** ^1^Allied Health Research Unit, St Vincent's Health Network Sydney (SVHNS), Sydney, NSW, Australia; ^2^Faculty of Health Sciences, Australian Catholic University, Sydney, NSW, Australia; ^3^Sydney School of Health Sciences, Faculty of Medicine and Health, The University of Sydney, Camperdown, NSW, Australia; ^4^The StrokeEd Collaboration, Ashfield, NSW, Australia; ^5^Alfred Health, Melbourne, VIC, Australia; ^6^Department of Neuroscience, Faculty of Medicine, Nursing & Health Sciences, Central Clinical School, Monash University, Melbourne, VIC, Australia; ^7^School of Clinical Medicine, University of New South Wales, Sydney, NSW, Australia; ^8^Department of Rehabilitation, St Vincent's Health Network Sydney (SVHNS), Sydney, NSW, Australia; ^9^Physiotherapy Department, Waikato Hospital, Hamilton, New Zealand; ^10^Arm's Reach Occupational Therapy, Bristol, United Kingdom; ^11^Harrison's Training, Wiltshire, United Kingdom; ^12^Centre for Behaviour Change, University College London, London, United Kingdom; ^13^Melbourne Medical School, The University of Melbourne, Parkville, VIC, Australia; ^14^Department of Rehabilitation, University of La Frontera, Temuco, Chile; ^15^Nursing Research Institute, St Vincent's Health Network Sydney, St Vincent's Hospital Melbourne & Australian Catholic University, Sydney, NSW, Australia

**Keywords:** telerehabilitation, stroke, behavior change, implementation, upper extremity (arm), occupational therapy, physiotherapy

## Abstract

**Background:**

Difficulty using the upper extremity in everyday activities is common after stroke. Constraint-induced movement therapy (CIMT) has been shown to be effective in both sub-acute and chronic phases of stroke recovery and is recommended in clinical practice guidelines for stroke internationally. Despite reports of equivalence of outcome when stroke rehabilitation interventions are delivered using telehealth, there has been limited evaluation of CIMT when using this mode of delivery. ReCITE will (a) evaluate the feasibility and acceptability of CIMT when delivered via telehealth to stroke survivors (TeleCIMT) and (b) explore therapists' experiences and use of an online support package inclusive of training, mentoring and resources to support TeleCIMT delivery in clinical practice.

**Methods:**

A prospective single-group, single blinded, study design with embedded process evaluation will be conducted. The study will be conducted at three outpatient services in Sydney, Australia. A multi-faceted therapist support package, informed by the Capabilities, Opportunity, Motivation- Behaviour model (COM-B), will be used to support occupational therapists to implement TeleCIMT as part of routine care to stroke survivors. Each service will recruit 10 stroke survivor participants (*n* = 30) with mild to moderate upper extremity impairment. Upper extremity and quality of life outcomes of stroke survivor participants will be collected at baseline, post-intervention and at a 4 week follow-up appointment. Feasibility of TeleCIMT will be evaluated by assessing the number of stroke participants who complete 80% of intensive arm practice prescribed during their 3 week program (i.e., at least 24 h of intensive arm practice). Acceptability will be investigated through qualitative interviews and surveys with stroke survivors, supporter surveys and therapist focus groups. Qualitative interviews with therapists will provide additional data to explore their experiences and use of the online support package.

**Discussion:**

The COVID-19 pandemic resulted in a rapid transition to delivering telehealth. The proposed study will investigate the feasibility and acceptability of delivering a complex intervention via telehealth to stroke survivors at home, and the support that therapists and patients require for delivery. The findings of the study will be used to inform whether a larger, randomized controlled trial is feasible.

## Introduction

Constraint-induced movement therapy (CIMT) is an intensive motor training approach used following stroke to overcome learned non-use of the upper limb (1, 2). CIMT involves three key components (1) high intensity, task orientated practice using the more affected arm for several hours a day for 2 to 3 weeks, (2) restraint of the unaffected arm using a mitt for up to 90% of waking hours to encourage use of the affected arm, and (3) use of a transfer package of monitoring mechanisms including behavioral contracts, a daily home diary, and daily homework to help participants generalize skills learned into daily life by using their affected arm more (3–5). High level evidence for CIMT exists, with statistically significant and clinically meaningful outcomes for people following stroke in both acute and chronic recovery stages (3, 6). CIMT is recommended as an effective intervention in clinical guidelines internationally for people with upper extremity impairment following stroke (1, 7, 8). The original model of CIMT required stroke survivors to participate in 6 h of daily task practice and to wear a mitt for 90% of waking hours (9). Since this trial, several different models of CIMT delivery have been used within trials which vary in intensity (2, 4, 5, 10–12), duration, group vs. one-on-one (13) and time post stroke (14), however the studies cited in guidelines have all been delivered face-to-face.

Adoption of CIMT has not been universal, and numerous barriers to delivery are cited in the literature (15–17) as well as patient-identified barriers to participation (18). Specifically, the barriers to patient participation include the time commitment and intensity of CIMT, while enablers to completing a CIMT program include support from therapists, carers/supporters and seeing improvements in arm function (19). Recent research has shown that patients (*n* = 40) receiving either in-person or virtual CIMT were “highly satisfied” with CIMT overall and found it only “moderately difficult” to participate in, based on survey data (20).

More broadly, stroke rehabilitation delivered via telehealth has been shown to attain similar outcomes to face-to-face delivery (21–23). While patients or participants are generally satisfied with this mode of delivery, the presence of family support may be a key variable influencing the success of telehealth interventions (24). The delivery of CIMT using telehealth may address patient barriers to attending the clinic to receive this intensive intervention, but may equally introduce further challenges or be found to be less effective. A previous study of patients completing face-to-face CIMT at home with family supporters and minimal therapist support (5 h total) showed no additional benefit compared to usual upper extremity therapy, suggesting that therapist support may play a key role in CIMT's effectiveness (25). Additionally, the complete transfer package was not used in that study, which may have been another factor contributing to the lack of treatment superiority (25). Other randomized controlled trials of home based CIMT have relied on specifically designed workstations and software (26–28) or gaming devices (29, 30) to deliver CIMT in the home. The use of such devices has shown promise, however may not be feasible to use in practice in health services servicing geographically isolated clients, such as those in regional or remote areas of Australia. What remains unknown is whether CIMT delivered via telehealth (TeleCIMT) can be implemented within usual care, using existing resources and remote therapist support and without the use of specialized equipment, other than a device with internet and video functionality.

The experience of telehealth from the perspective of clinicians has been less favorable (31). Delivering an intervention using telehealth changes workflows and processes, and interventions need to be adapted (32). This raises many barriers, principally time, skills and resources, with clinicians generally preferring to deliver interventions face-to-face (24). The use of behavior change theory can support the implementation of complex health interventions including CIMT (33). A previous study by members of this research group, the ACTIveARM project (34) (published thesis), used a multimodal behavior change intervention targeted at clinicians to successfully increase face-to-face CIMT delivery in public health settings. A behavior change intervention including education of clinicians, provision of resources and support to help them adapt their practice, has the potential to reduce identified barriers to telerehabilitation.

The COVID-19 pandemic impacted the delivery of intensive face-to-face rehabilitation interventions, including CIMT. During this period, the TeleCIMT International Development (TIDE) group of occupational therapists, physiotherapists and researchers from Australia, United Kingdom and New Zealand developed resources to support CIMT delivery via telehealth (TeleCIMT) (35). The free resources for therapists and stroke survivors include educational webinars and videos, step-by-step CIMT practice booklets and exercise libraries (35). While these resources are informed by evidence, their use in clinical practice, the safety of patient participants to complete TeleCIMT, and experiences of TeleCIMT for therapists, stroke survivors and their supporters, have not yet been evaluated.

Therefore the Remote Constraint Induced Therapy of the upper Extremity (ReCITE) pilot will investigate the feasibility and acceptability of CIMT via telehealth, within routine practice. In relation to feasibility, the study will explore;

- Whether it is feasible and safe for community stroke survivors to complete at least 80% of a 3 week TeleCIMT program at home (i.e., 24 h of intensive upper extremity therapy) with remote therapist support
- The clinical outcomes of delivery of CIMT via telehealth

In relation to acceptability, the study will explore:

- The acceptability of TeleCIMT delivery to stroke survivors and their supporters
- Whether therapy teams adopt and deliver CIMT programs via telehealth after receiving a TeleCIMT online therapist support package and the barriers and enablers to TeleCIMT delivery in practice.

## Method

### Design

**Figure 1:**
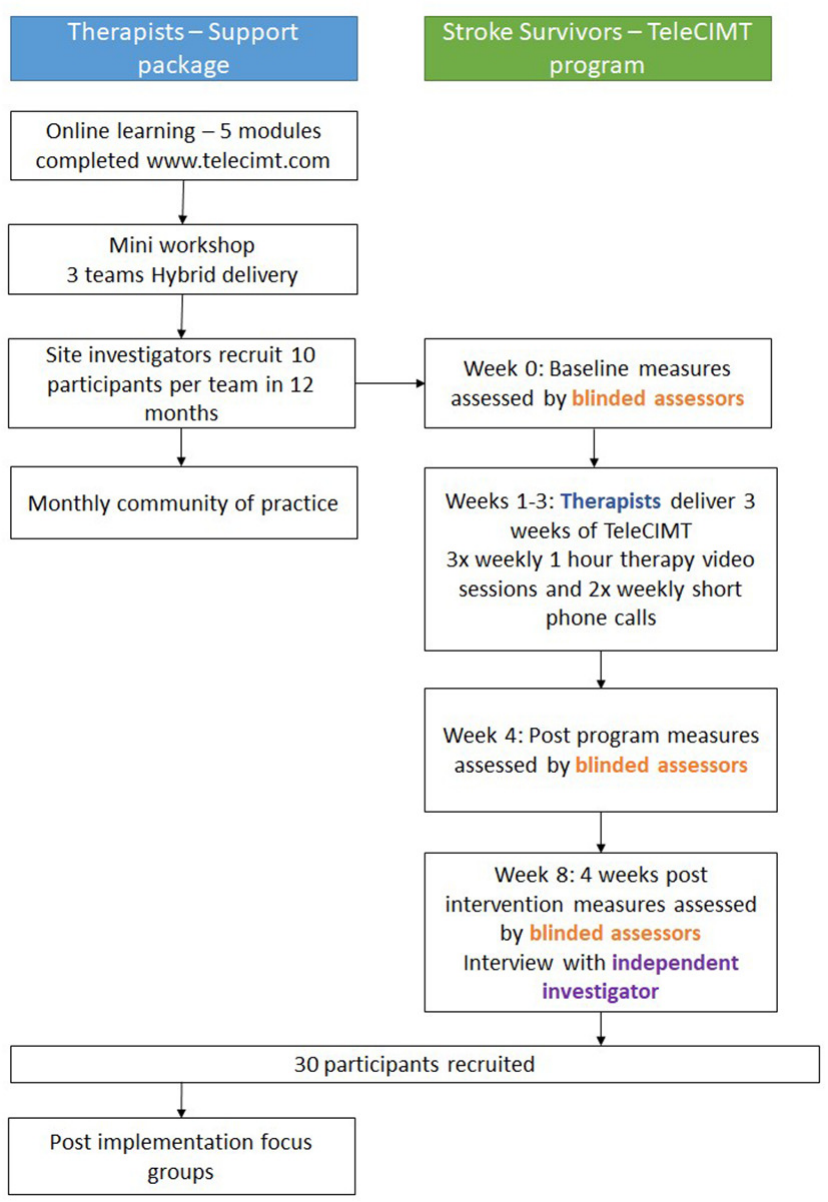
ReCITE study flowchart.

A prospective, single blinded, pre-test post-test study design will be used. To support rigor in this non-randomized design, we will use blinded outcome assessment and embedded qualitative evaluation. The study design is presented in Figure 1, guided by the Consolidated Standards of Reporting Trials (CONSORT) statement (36). The study is reported according to Standard Protocol Items: Recommendations for Interventional Trials (SPIRIT) (37), Standards for Reporting Implementation Studies (StaRI) Statement (38) and the TIDieR framework for describing evidence-based interventions (39).

The study is registered with the Australian New Zealand Clinical Trials Registry (ACTRN12622000082707). Any changes to the trial protocol will be reported. This study was approved by the St Vincent's Hospital and South Western Sydney Local Health District Human Research Ethics Committees (2021/ETH01131).

### Setting and participants

This study will involve a sample of three selected public hospital outpatient rehabilitation services within metropolitan Sydney, Australia. Services typically offer publicly funded outpatient occupational therapy and physiotherapy sessions to sub-acute and chronic stroke survivors for 6 to 8 weeks post discharge from hospital.

There will be two groups of participants: therapists and stroke survivors.

#### Therapist participants

Therapist participants (*n* = 12) will be occupational therapists currently employed at one of the three outpatient rehabilitation services and involved in delivery of the TeleCIMT program.

#### Stroke survivor participants

All stroke survivor participants who present for outpatient upper extremity rehabilitation at participating sites will be screened by therapist site investigators during routine therapy. Eligibility criteria are outlined in Box 1. All participants will have been discharged home from hospital and therefore will be either in the subacute or chronic phase post stroke. There are no restrictions placed on maximum time post stroke for study inclusion. Once screened and deemed eligible for study inclusion, potential participants will be provided with an information sheet and consent form. Once consented, baseline assessments will be scheduled with trained, blinded assessors and completed prior to intervention commencement.

Box 1 **Inclusion criteria for stroke survivor participants.**

1) 18 years or older.
2) Confirmed diagnosis of stroke resulting in mild to moderate upper extremity impairments as measured by the Motor Assessment Scale item 8 (40).
3) Meet CIMT upper limb criteria including recovery of some active finger extension in at least two digits, some active thumb extension and some active wrist extension in the affected upper limb (1, 2, 41). Participants must have at least 10° of active wrist extension, at least 10° of thumb abduction/extension, and at least 10° of extension in at least 2 additional fingers. These movements must be repeated 3 times in 1 min.
4) Medically stable.
5) Able to participate in intensive rehabilitation at least 2 h per day for 3 weeks.
6) Able to read, speak and understand English.
7) Aphasia severity rating (ASR) score of at least three (42).
8) Have adequate cognition to participate in a CIMT program with the assistance of a supporter as determined by a Montreal Cognitive Assessment (MoCA) score of 18 to 25 or with or without a supporter as determined by a MoCA score of 26 and above (range 0–30) (43).
9) Sufficient reading acuity as measured by the Occupational Therapy Adult perceptual screening test—visual acuity item) (44).

### Intervention

#### Therapist support package

The ReCITE therapist support package will be tailored to each therapy team, and support them to deliver TeleCIMT. The therapist support package development was informed by the Theoretical Domains Framework (45) and Behavior Change Wheel (46), and will be described elsewhere. The package was refined with input from occupational therapy managers and occupational therapists during a focus group. Table 1 provides details of support package components in accordance with the TIDieR checklist (39). In summary, the package includes four components:

**Table 1:** Description of multicomponent therapist support package delivered to teams of occupational therapists.

**Intervention component**	**Rationale/barriers addressed**	**Mode of delivery**	**Delivered by whom, To whom**	**When/how often**
Online education package (2–3 h in duration) (pre-recorded presentations, videos and written resources/slides)	To increase content knowledge and skills.	Online via www.telecimt.com; individual	Delivered to: Occupational Therapists Delivered by: Pre-recorded education sessions by TeleCIMT development team	Once at beginning of support package Can be used to train any new/ additional therapists as needed
Online workshop (3 h)	To increase clinician confidence and provide opportunity to practice skills. To act as an incentive/motivator to complete online sessions. Use questionnaire prior to workshop to measure knowledge after online training modules and to tailor workshop to local needs.	Hybrid (facilitators online, therapists face to face in teams)	Delivered to: Occupational Therapists Delivered by: Facilitated by Chief Investigator (LJC) with support from Associate Investigators NF and TIDE team	After completion of the online training, before beginning TeleCIMT therapy sessions
Teleconference community of practice (COP) with site investigators	To address barriers related to practical social support, action planning and problem solving, support needs of new clinicians, assist with screening, eligibility and getting started with CIMT. Online forum for issues required between COP—to be reviewed before each COP.	Via Microsoft teams for ReCITE trial	Delivered to: Occupational Therapists Delivered by: Facilitated by Chief Investigator (LJC) with support of Associate Investigators NF and TIDE team	Monthly
Provision of resources to support TeleCIMT delivery	To make delivery of TeleCIMT in clinical practice more feasible	Electronic documents available and downloadable online Via www.telecimt.com	Delivered to: Occupational Therapists Delivered by: Resources produced by TeleCIMT development team	Can be used by therapists as needed during each TeleCIMT program

Education: Therapists complete five online, self-directed learning modules about TeleCIMT theory via the www.telecimt.com website (therapist learning resources).

Skills and knowledge consolidation: After completing the online training, a 3 h virtual workshop will be delivered to therapists in their work environment. Therapists will practice delivering CIMT via telehealth.

Patient and therapist resources: Resources for delivering and recording TeleCIMT are available from the www.telecimt.com website. Therapists will select and download the resources required for their team to deliver TeleCIMT.

Community of practice: A monthly virtual community of practice will be facilitated by the research team, supporting therapists' involved in the study with troubleshooting, and discussion of barriers and enablers. Microsoft teams chat will allow therapists to raise (de-identified) problems and ask questions, and provide peer support between monthly meetings.

#### Evidence-based intervention—TeleCIMT

The evidence-based intervention to be implemented into practice is a 3 week CIMT program delivered via telehealth (TeleCIMT) to stroke survivors. This intervention will be delivered after therapists' receive the therapist support package. Stroke survivor participants will participate in 3 weeks of TeleCIMT delivered by an occupational therapist. The TeleCIMT model was developed by JB, AM, AK, and LJC and is based on current Australian stroke clinical guideline recommendations for CIMT delivery (1): a minimum of 2 h of intensive practice per day [including shaping and functional tasks (2)], 6 h of mitt wearing per day and a transfer package to support program adherence and behavior change by stroke participants for at least 2 weeks (1). The TeleCIMT program uses shaping and functional task practice libraries (www.telecimt.com) and increased involvement of a carer or supporter to deliver a program remotely with reduced, and solely virtual, therapist input.

Shaping is a training method based on principles of behavior training in which tasks are completed in a series of 10 timed trials (30 s to 2 min per timed trial), with feedback provided about performance immediately after each trial (47). Tasks are selected that target a participant's specific motor impairments and movements that have the greatest potential to improve and are set at a level that is challenging but achievable for the participant (48). Tasks are made incrementally more challenging (“shaped”) throughout the participant's program and coaching and encouragement are provided by the therapist throughout all shaping trials (48).

Task practice involves the completion of functional activities (e.g., folding laundry) using only the affected upper limb for 15 to 20 min per activity. The complexity of the prescribed functional tasks are progressed throughout the program (48).

Shaping and functional tasks selected for each program will be individualized to the participant's goals and interests and motor abilities; and will use items already available within the participant's home to support training activities wherever possible.

Participants will receive three, 1 h intensive therapy sessions (shaping and functional tasks) per week via video call, and two brief, 30 min telephone or video calls 2 days per week (total therapist contact 5 days per week for 3 weeks). The shorter telephone or video calls will be used on alternate weekdays to complete components of the transfer package including providing encouragement and positive feedback, to monitor practice completed and to progress practice activities. Outside of these structured times, participants will be required to follow the CIMT program outlined by their treating therapist within their TeleCIMT workbook and complete a minimum of 2 h of active practice per day with or without the support of a carer. Table 2 outlines the structure and components of the TeleCIMT program using the Template for Intervention Description and Replication (TIDIeR) checklist (39).

**Table 2:** TeleCIMT intervention description using the Template for Intervention Description and Replication (TIDIeR) checklist (39).

	**TeleCIMT program (35)**
Why	CIMT is a strongly recommended intervention to improve arm recovery after stroke in the Australian and New Zealand Clinical Guidelines for Stroke Management but CIMT is underutilized in practice. Delivering CIMT via telehealth (TeleCIMT) may enhance program reach to more stroke survivors including those residing in regional and remote communities.
What procedures	Participants will receive a 3 week TeleCIMT program as part of their routine care, comprising: • Intensive graded practice using their affected arm for at least 2 h per day including both 1 h of shaping tasks and 1 h of functional task practice, 5 days per week. • A mitt restraint worn on the non-affected upper limb to encourage use of the affected arm for at least 6 h per day, 5 days per week. • A transfer package to encourage generalization of use of the affected arm in daily life. The transfer package will include review of practice, a daily diary and problem solving challenges with using the affected arm in daily activities with a therapist 5 days per week, a behavioral contract for both participant and supporter, signed at program commencement to encourage adherence and establish guidance for safe use of the mitt and a daily schedule to guide mitt on and off times.
What materials	Therapists have access to the TeleCIMT website (www.telecimt.com) that includes the following patient related resources: • TeleCIMT assessment form. • TeleCIMT preparation pack to assist with planning goals and activities during a TeleCIMT program, mitt on and mitt off activities, a daily schedule, a behavioral contract for both the participant and their supporter and tips for supporters in their role assisting with a program. • TeleCIMT preparation videos. • TeleCIMT program pack which includes daily records of practice, a brief daily diary, program activities and recommended progressions. • Shaping and functional task practice libraries to assist with selecting and progressing program exercises. • Each team provided with 10 mitt restraints and 1 Microsoft webcam to loan to participants if needed to support video calls. Pocket Wifi device will be loaned to stroke participants who do not have access to the internet and this is the only reason for exclusion from the study. • Items for shaping and functional tasks will be items available in the participant's home or low cost items from the therapist's clinical resources. Items for therapy will be based on each participant's individual goals for the program.
Who provided	TeleCIMT will be delivered by tertiary trained occupational therapists employed in public health outpatient services who consent to participate in the study. All who consent will receive the therapist support package to support them in delivering TeleCIMT.
How	The TeleCIMT program will be delivered as part of routine care, remotely via video and telephone calls. Program resources such as the preparation pack, program pack, mitt and camera may be provided to the participant during a face to face session prior to program commencement or sent via post.
Where	TeleCIMT will be delivered remotely to participants in their homes.
When and how much	Participants will receive one program with therapist input 5 days per week for 3 weeks. 1 h therapy sessions will be provided to participants 3 days per week via video call. On alternate days (i.e., 2 days per week), telephone calls will be completed to review independent practice, progress activities and complete an online transfer package (~15 to 20 min).
Tailoring	This is a tailored intervention. Program activities will be set based on the patient's goals, upper limb function, key upper extremity impairments and support available to them at home. An additional 30 min of homework practice may also be prescribed for participants who are more inactive. The core elements that all participants should complete are at least 2 h of practice per day involving both shaping tasks and functional task practice activities that are regularly progressed, 6 h of mitt wear on the non-affected arm to encourage use of the affected arm in daily activities and a telephone review of program progress to support program adherence, problem solving and generalization of use of the affected arm in daily life.

### Data collection and outcome measures

Feasibility and acceptability of delivering TeleCIMT in routine practice will be investigated in depth using several outcomes.

#### Therapist outcomes

Feasibility of recruitment will be evaluated by measuring the number of stroke survivors recruited for TeleCIMT at each site over the period of 12 months, as a proportion of those deemed eligible for CIMT. Pace of recruitment is anticipated to be a minimum of 10 participants per site per year to indicate feasibility for a larger randomized controlled trial to be conducted.

Feasibility of delivering the therapist support package will be evaluated by measuring the number/proportion of therapist participants completing each learning module, and download rates of TeleCIMT resources using Google analytic web usage data. Participation rates in the monthly community of practice will also be evaluated for each of the therapy teams.

Perceived barriers to, and enablers of behavior change and TeleCIMT delivery will be captured using the self-evaluation Capabilities, Opportunities, Motivation- Behaviour (COM-B) questionnaire (50) at three time points; prior to delivery of the support package, after completion of online learning and after the mini-workshop. TeleCIMT knowledge will be assessed using an online knowledge quiz following completion of online learning modules.

Post-implementation, focus group interviews will be conducted with site therapists involved in the delivery of two or more TeleCIMT programs, using an interview schedule informed by the reach, effectiveness, adoption, implementation and maintenance (RE-AIM) framework (51). Focus groups will explore TeleCIMT feasibility and acceptability, and barriers and enablers to delivery perceived by clinicians, with respect to their clinical setting. The focus groups will be facilitated by an experienced qualitative researcher within the research team who is independent of all participating health services.

#### Stroke survivor outcomes

##### Feasibility outcomes

Program fidelity will be determined by evaluating process outcomes, including (a) number of sessions completed, supervised and unsupervised, as a proportion of planned sessions, (b) repetitions and amount of practice time recorded during supervised and unsupervised sessions. Delivery of TeleCIMT in practice will be deemed feasible if stroke survivor participants complete at least 80% of intensive arm practice during their 3 week TeleCIMT program (i.e., at least 24 h of intensive arm practice). Amount of practice will be measured as the number of minutes of practice completed per day, as reported by each stroke survivor and/or their supporter during their daily telephone/video call.

Acceptability will be determined by qualitative semi-structured interviews, conducted individually with up to 20 TeleCIMT participants by telephone, video call or in person, dependent on participant preference, within 1 month of completing TeleCIMT. Interviews will be recorded and transcribed verbatim to explore stroke survivors' experiences of completing TeleCIMT. Acceptability will also be determined quantitatively using the participant opinion survey (POS) (20).

##### Clinical outcomes

Upper limb and quality of life outcomes will be measured prior to commencement of TeleCIMT (week 0), immediately post TeleCIMT program (week 4) and 1 month follow-up (week 8) by trained therapists, blinded to the intervention. Change in upper extremity function will be assessed using five standardized, reliable and valid assessments: the Action Research Arm Test (ARAT) (52), Box and Block Test (BBT) (53), Nine Hole Peg Test (NHPT) (54), Grip Strength (55), and the Motor Activity Log (MAL-30) (56, 57). Quality of life outcomes will also be collected using the EuroQOL 5 dimensions, 5 level (EQ 5D 5L) (58), and Stroke Impact Scale-16 (SIS-16) (59).

##### Supporter outcomes

Supporters who provide assistance to participants during their TeleCIMT program will be invited to participate in an online survey following program completion to assess TeleCIMT acceptability to supporters. The survey consists of demographic questions and the generic theoretical framework of acceptability (TFA) questionnaire (60), tailored to the role of the supporter in a TeleCIMT program. The TFA questionnaire consists of seven component constructs of acceptability and an overall acceptability measure.

##### Safety outcomes for TeleCIMT

The number and type of adverse events experienced by participants during TeleCIMT, such as illness, extreme fatigue, muscle soreness, shoulder pain or injuries, trips (a near miss but did not fall to the ground and no injury sustained) and falls (fall to the ground, with or without sustaining an injury) will be recorded after each contact with a blinded assessor or therapist. Therapists delivering TeleCIMT and blinded assessors completing outcome measures will report any adverse events reported or experienced by stroke survivors to the site principal investigator and chief investigator. They will record whether or not the event appears to be caused by / associated with TeleCIMT or other concurrent activities. A register of any adverse events will be maintained and any serious adverse events reported to the Human Research Ethics Committee. Dependent on the nature of the event, escalation may also include referral to a general practitioner, hospital and/or discontinuation or suspension of the intervention (TeleCIMT).

### Data management

Study data will be collected and managed using Research Electronic Data Capture (REDCap) tools hosted at St Vincent's Health Network Sydney. Site investigators will enter screening data, demographic information and CIMT program data for all included participants. De-identified screening information will also be collected by site investigators to explore reasons why potential participants were not eligible for, or refused study participation.

Blinded assessors will enter outcome measurement data following each assessment timepoint. The chief investigator and senior research officer will regularly review completeness of data entered by therapists and blinded assessors.

### Data analysis

#### Sample size for TeleCIMT participants

We will recruit 30 TeleCIMT participants (up to 10 stroke survivors per site). A sample size of 29 stroke survivors is required to assess the feasibility of 90% of participants completing at least 24 h (80% of planned hours) of intensive practice during their TeleCIMT program with precision (half-width 95% confidence interval) of 11% (61). Outcomes will be reported with 95% confidence intervals and analyses will assume a two-sided level of significance of *p* < 0.05.

#### Feasibility outcomes

Descriptive statistics (mean [SD] or number [%]) will be used to analyse the proportion of participants recruited from those eligible for TeleCIMT, therapist adherence to the therapist support package, including completion of online education modules, download rates, and participation in the community of practice and changes in perceived barriers and enablers on the COM-B questionnaire. The proportion of participants completing 80% of a TeleCIMT program (at least 24 h of intensive upper extremity practice) will be analyzed and reported using descriptive statistics to explore TeleCIMT participant program feasibility, adherence and fidelity. The TeleCIMT recruitment will be considered feasible if 75% of eligible stroke participants are enrolled in the study and each team can recruit 10 stroke participants over a period of 12 consecutive months.

#### Upper extremity outcomes

Outcomes for learned non-use (MAL-30) and upper extremity outcomes (NHPT, ARAT, BBT, and grip strength) will be compared using paired *t*-tests (pre-post TeleCIMT program) and 95% confidence intervals, and linear mixed effect models (multiple time points) or non-parametric methods if data are not normally distributed. The proportion of participants achieving the minimal clinically important difference (MCID) post TeleCIMT will be analyzed using mixed effects logistic regression for the MAL-30 (1.0) (62), ARAT [12 points for acute stroke (63), and 5.7 points for chronic stroke (64)], and grip strength (62).

#### Quality of life outcomes

EQ5D5L raw scores will be converted to a utility-based index based on an Australian population (65). Health-related quality of life scores from the EQ5D5L and SIS-16 will be analyzed using paired *t*-tests (pre-post) and 95% confidence intervals, and linear mixed effect models (multiple time points). The proportion of participants achieving the MCID on the SIS-16 (9.4–14.1 points) (66) will be analyzed using mixed effects logistic regression. The relationship between the results from the EQ5D5L and the SIS-16 will be evaluated using Spearman bivariate correlation analysis.

#### Acceptability outcomes

Responses by stroke survivors to the POS and supporters to the TFA questionnaire will be analyzed descriptively.

**Figure 2:**
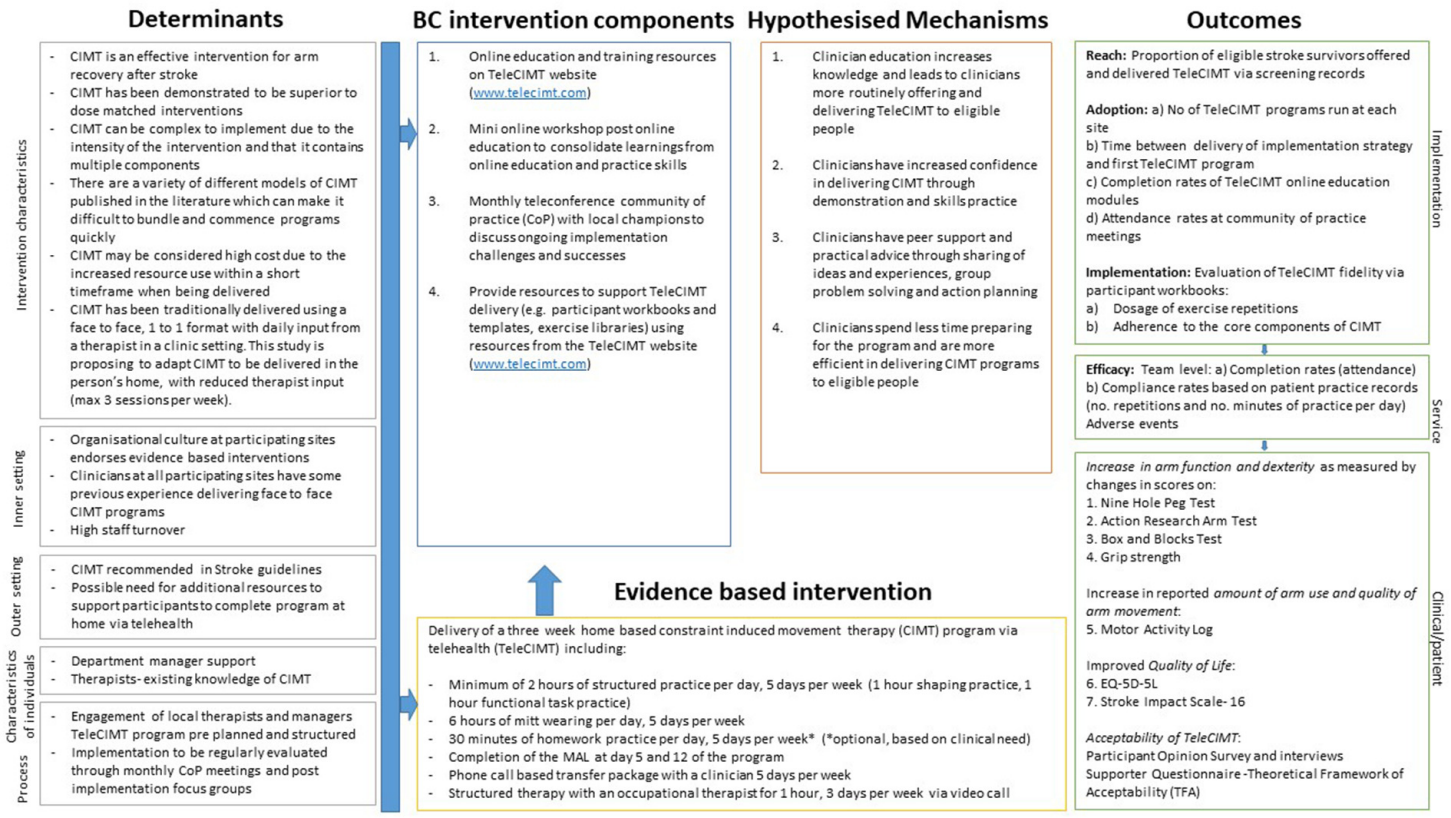
Implementation research logic model for ReCITE study (49).

Transcripts from individual interviews with stroke survivors and focus groups with therapists will undergo thematic analysis, using NVIVO software for data management. Focus group interviews will be deductively mapped to the RE-AIM framework. Tables will be generated to summarize team experiences of barriers and enablers to TeleCIMT delivery within clinical practice. Stroke survivor interviews will be coded using an inductive thematic analysis approach. Triangulation of data will be used to ensure consistency of coding and categories. Peer examination will be used to ensure the trustworthiness of the data collection and analysis (67). Figure 2 presents the logic model showing how therapist support package components are hypothesized to influence the outcomes of the study.

Summaries of the results will be presented to the services involved, and shared on the TIDE website. Consumers involved in the development of resources will also be provided with a summary of results from the study.

## Discussion

This study will present findings of the feasibility of TeleCIMT in an Australian context without the need for specialist devices other than a device with internet access and video capabilities. Distance from specialist rehabilitation services in Australia is an issue for many stroke survivors (68). In addition, the COVID-19 pandemic resulted in a rapid transition to delivering telehealth, however, research on the safety, feasibility and acceptability of delivering complex interventions via telehealth, and the support that therapists and patients require, has not been previously explored (69). Upper extremity outcomes will be evaluated and qualitative feedback from therapists, stroke survivors and supporters on TeleCIMT will explore these factors.

The intensive and complex nature of CIMT may not lend itself to remote delivery. Reduced therapist input and increased reliance on a support person, for the majority of stroke survivors (especially those with a cognitive impairment), to complete a program may impact on the feasibility and acceptance of TeleCIMT. Therefore, understanding of acceptance and feasibility is essential to explore if TeleCIMT can be delivered successfully, or if further adjustments need to be made to support therapists and participants with remote delivery of CIMT. If this study shows that TeleCIMT is acceptable and feasible, this group will seek to perform a larger, randomized controlled trial. This pilot feasibility study is not sufficiently powered to report on efficacy. We also acknowledge that the varied time post-stroke of participants may increase the variability of outcomes.

## Conclusions

The ReCITE study will explore the feasibility of TeleCIMT delivery to stroke survivors in routine practice. The study will also evaluate the safety and acceptability of TeleCIMT from the perspectives of therapists delivering TeleCIMT and stroke survivors receiving the intervention. These results will be used to evaluate if a larger randomized controlled trial is feasible, and help determine sample size calculations.

## Data availability statement

The original contributions presented in the study are included in the article/supplementary material, further inquiries can be directed to the corresponding author/s.

## Ethics statement

The studies involving human participants were reviewed and approved by St Vincent's Hospital Human Research Ethics Committee. The patients/participants provided their written informed consent to participate in this study.

## Author contributions

LJC, AMc, and SM conceptualized the study with input from NL regarding the study design. LJC, AMc, CS, AK, JB, AMe, SF, AD, and SM received funding to conduct this study. LJC and EH designed the multifaceted therapist support package with NF, NL, AMc, CS, AK, and SM. JB, AMe, AK, and LJC designed the TeleCIMT intervention. LC contributed to the design of the data analysis plan. All authors contributed to the design of the study protocol, manuscript draft, and provide consent for the final manuscript submission. All authors contributed to the article and approved the submitted version.

## Funding

This study was supported by a Stroke Foundation Early Career Seed Grant and St Vincent's Clinic Foundation Multidisciplinary Research Grant. NL was supported by a fellowship from the Heart Foundation (Australia), GNT102055. AD was supported by a University of La Frontera Research Project grant (Chile), DIUFRO 21/077.

## Conflict of interest

The authors declare that the research was conducted in the absence of any commercial or financial relationships that could be construed as a potential conflict of interest.

## Publisher's note

All claims expressed in this article are solely those of the authors and do not necessarily represent those of their affiliated organizations, or those of the publisher, the editors and the reviewers. Any product that may be evaluated in this article, or claim that may be made by its manufacturer, is not guaranteed or endorsed by the publisher.

## Abbreviations

ARAT: Action Research Arm Test
BBT: Box and Block Test
COM-B, Capability, Opportunity: Motivation-Behaviour model
CIMT: Constraint induced movement therapy
EQ 5D 5L, EuroQOL 5 dimensions: 5 level
MCID: minimal clinically important difference
MAL-30: Motor Activity Log
NHPT: Nine Hole Peg Test
SIS-16: Stroke Impact Scale-16
TDF: Theoretical Domains Framework
TFA: Theoretical Framework of Acceptability
POS: Participant Opinion Survey
ReCITE: Remote Constraint Induced Therapy of the upper Extremity.
